# Clinical significance of circulating miR-25-3p as a novel diagnostic and prognostic biomarker in osteosarcoma

**DOI:** 10.18632/oncotarget.16498

**Published:** 2017-03-23

**Authors:** Tomohiro Fujiwara, Koji Uotani, Aki Yoshida, Takuya Morita, Yutaka Nezu, Eisuke Kobayashi, Akihiko Yoshida, Takenori Uehara, Toshinori Omori, Kazuhisa Sugiu, Tadashi Komatsubara, Ken Takeda, Toshiyuki Kunisada, Machiko Kawamura, Akira Kawai, Takahiro Ochiya, Toshifumi Ozaki

**Affiliations:** ^1^ Department of Orthopedic Surgery, Okayama University Graduate School of Medicine, Dentistry, and Pharmaceutical Sciences, Okayama, Japan; ^2^ Center of Innovative Medicine, Okayama University Hospital, Okayama, Japan; ^3^ Division of Molecular and Cellular Medicine, National Cancer Center Research Institute, Tokyo, Japan; ^4^ Department of Musculoskeletal Oncology, National Cancer Center Hospital, Tokyo, Japan; ^5^ Division of Pathology and Clinical Laboratories, National Cancer Center Hospital, Tokyo, Japan; ^6^ Department of Intelligent Orthopaedic System, Okayama University Graduate School of Medicine, Dentistry, and Pharmaceutical Sciences, Okayama, Japan; ^7^ Department of Medical Materials for Musculoskeletal Reconstruction, Okayama University Graduate School of Medicine, Dentistry, and Pharmaceutical Sciences, Okayama, Japan; ^8^ Department of Hematology, Saitama Cancer Center, Saitama, Japan

**Keywords:** microRNA, liquid biopsy, osteosarcoma, biomarker, prognosis

## Abstract

**Background:**

Emerging evidence has suggested that circulating microRNAs (miRNAs) in body fluids have novel diagnostic and prognostic significance for patients with malignant diseases. The lack of useful biomarkers is a crucial problem of bone and soft tissue sarcomas; therefore, we investigated the circulating miRNA signature and its clinical relevance in osteosarcoma.

**Methods:**

Global miRNA profiling was performed using patient serum collected from a discovery cohort of osteosarcoma patients and controls and cell culture media. The secretion of the detected miRNAs from osteosarcoma cells and clinical relevance of serum miRNA levels were evaluated using *in vitro* and *in vivo* models and a validation patient cohort.

**Results:**

Discovery screening identified 236 serum miRNAs that were highly expressed in osteosarcoma patients compared with controls, and eight among these were also identified in the cell culture media. Upregulated expression levels of miR-17-5p and miR-25-3p were identified in osteosarcoma cells, and these were abundantly secreted into the culture media in tumor-derived exosomes. Serum miR-25-3p levels were significantly higher in osteosarcoma patients than in control individuals in the validation cohort, with favorable sensitivity and specificity compared with serum alkaline phosphatase. Furthermore, serum miR-25-3p levels at diagnosis were correlated with patient prognosis and reflected tumor burden in both *in vivo* models and patients; these associations were more sensitive than those of serum alkaline phosphatase.

**Conclusions:**

Serum-based circulating miR-25-3p may serve as a non-invasive blood-based biomarker for tumor monitoring and prognostic prediction in osteosarcoma patients.

## INTRODUCTION

The lack of useful biomarkers is one of the most important clinical problems of bone and soft tissue sarcomas. Osteosarcoma, the most common primary bone malignant tumor arising in children and young adults [1, 2], is no exception; the detection of the primary tumor or tumor relapse has generally relied on imaging methods such as X-ray, computed tomography (CT), positron emission tomography (PET)-CT, magnetic resonance imaging (MRI), and scintigraphy. Along with the development of surgical techniques [3, 4] and multi-agent chemotherapy [5, 6], patient prognosis has gradually improved over the past 30 years. Current multi-chemotherapeutic regimens, including neoadjuvant and adjuvant doxorubicin, cisplatin, methotrexate, and/or ifosfamide have maintained 5-year overall survival rates at approximately 60–80% [5–9]. However, for patients who present with local recurrence or metastasis, outcomes are far worse, with survival rates below 30% within 5 years of diagnosis [9, 10]. Furthermore, the histological response to neoadjuvant chemotherapy is the most dependable and reproducible prognostic indicator of the probability of disease-free survival [5–9]. Thus, development of methodologies for real-time monitoring of drug response and early detection of recurrence or metastasis will further improve patient prognosis. Currently, less complex monitoring using patient blood has not been developed. Alkaline phosphatase (ALP), a known serum-based tumor marker of osteosarcoma, sometimes provides false positives since ALP is generally elevated in children and affected by organ damage. Thus, the development of highly sensitive, specific, and minimally invasive biomarkers that can be used to detect and monitor tumor burden of tumors is the most important challenge for osteosarcoma management.

Evidence of microRNA (miRNA) dysregulation in malignant tumors has emerged in recent years. miRNAs are small non-coding RNA molecules that modulate the expression of multiple target genes and play important roles in various physiological and pathological processes, such as development, differentiation, cell proliferation, apoptosis, organogenesis, and homeostasis [11, 12]. After the discovery of miRNA in 1993 [13], its importance in malignant disease was suggested in 2004 when miRNA genes were found to be specifically deleted in leukemia [14, 15]. Subsequent reports have demonstrated that miRNAs are dysregulated in many malignant tumors, and these miRNAs can initiate carcinogenesis or drive progression [15, 16]. Following the discovery of miRNA dysregulation in cancer, miRNA dysregulation in osteosarcoma cells and/or tissues was reported since 2009 [17–44].

Tumor cells have recently been demonstrated to secrete miRNAs into the circulation [45]. Despite the presence of RNase activity in human blood, data have demonstrated that serum miRNAs remain stable under protection by exosomes or argonaute 2 [46, 47]. Analysis of circulating miRNA levels in patient blood presents a novel approach for diagnostic cancer screening or monitoring. Lawrie *et al*. was the first to report that tumor-associated miRNA levels in patient serum were higher than those in healthy individuals, indicating that circulating miRNAs may be used as biomarkers to monitor cancer cells [48]. To date, differential expression of circulating miRNA has been reported in cancers of the breast, lung, stomach, liver, kidney, bladder, prostate, and ovaries, among others [49–51]. This evidence on the potential of circulating miRNAs in patient blood is transforming the field of clinical biomarkers. However, comprehensive profiling studies of circulating miRNAs in osteosarcoma are limited and a consistent diagnostic signature for circulating miRNAs is not available.

In this study, we performed global miRNA screening in patient serum, following a validation study of *in vitro* and *in vivo* analysis. In addition, we evaluated whether the specified miRNA could monitor osteosarcoma and its drug response during multimodal treatment including chemotherapy and surgery.

## RESULTS

### Global miRNA expression analysis of serum in osteosarcoma patients, age-matched controls, and healthy individuals

**Figure 1:**
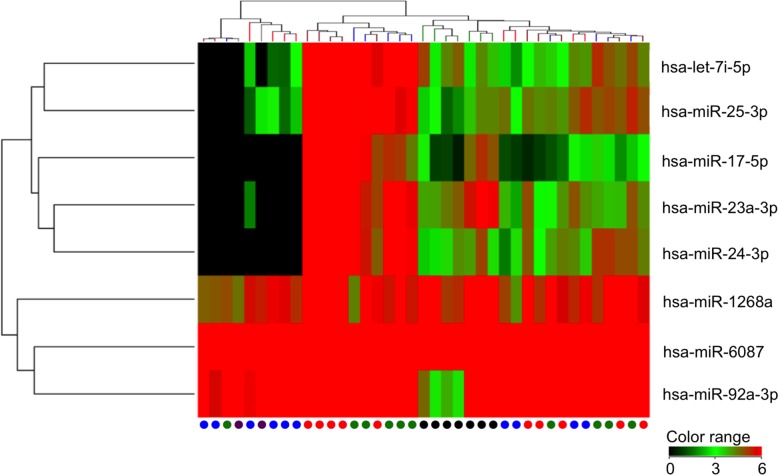
Hierarchical clustering of serum samples and culture media Heatmap showing expression of candidate miRNAs that are upregulated in the serum of oseteosarcoma patients compared with controls and are expressed in the culture media of osteosarcoma cell lines. Serum from osteosarcoma patients at preoperative state and postoperative state are indicated with red and purple, respectively. Serum from patients with benign tumors and healthy individuals are indicated with green, and blue circles, respectively. Culture media from osteosarcoma cell lines are indicated with black circles.

This study was designed as an initial screening phase and a subsequent validation phase. The initial global miRNA screening included the serum samples from 10 osteosarcoma patients, 10 age-matched non-osteosarcoma patients, 10 healthy volunteers, and the culture medium from seven osteosarcoma cell lines (Supplementary Figure 1). The mean age of the osteosarcoma patients was 23.4 ± 13.4 years and that of the benign tumor patients was 20.0 ± 12.4 years; no significant difference in age between the two groups was observed (*P* = 0.56). The healthy volunteers were adults with a mean age of 32.4 ± 10.2 years. In the combined pool of human miRNAs, 236 serum miRNAs were identified to be highly expressed (>1.5 fold-change) in the serum of osteosarcoma patients compared with benign tumor patients and healthy volunteers. Among these miRNAs, eight miRNAs overlapped with the secretory miRNAs detected in the culture medium of the seven osteosarcoma cell lines (SaOS2, U2OS, HOS, MNNG/HOS, 143B, MG63, and HuO9) (Figure 1, Supplementary Table 1).

### Upregulated expression of miR-25a-3p and miR-17-5p in osteosarcoma cells and cell culture media

**Figure 2:**
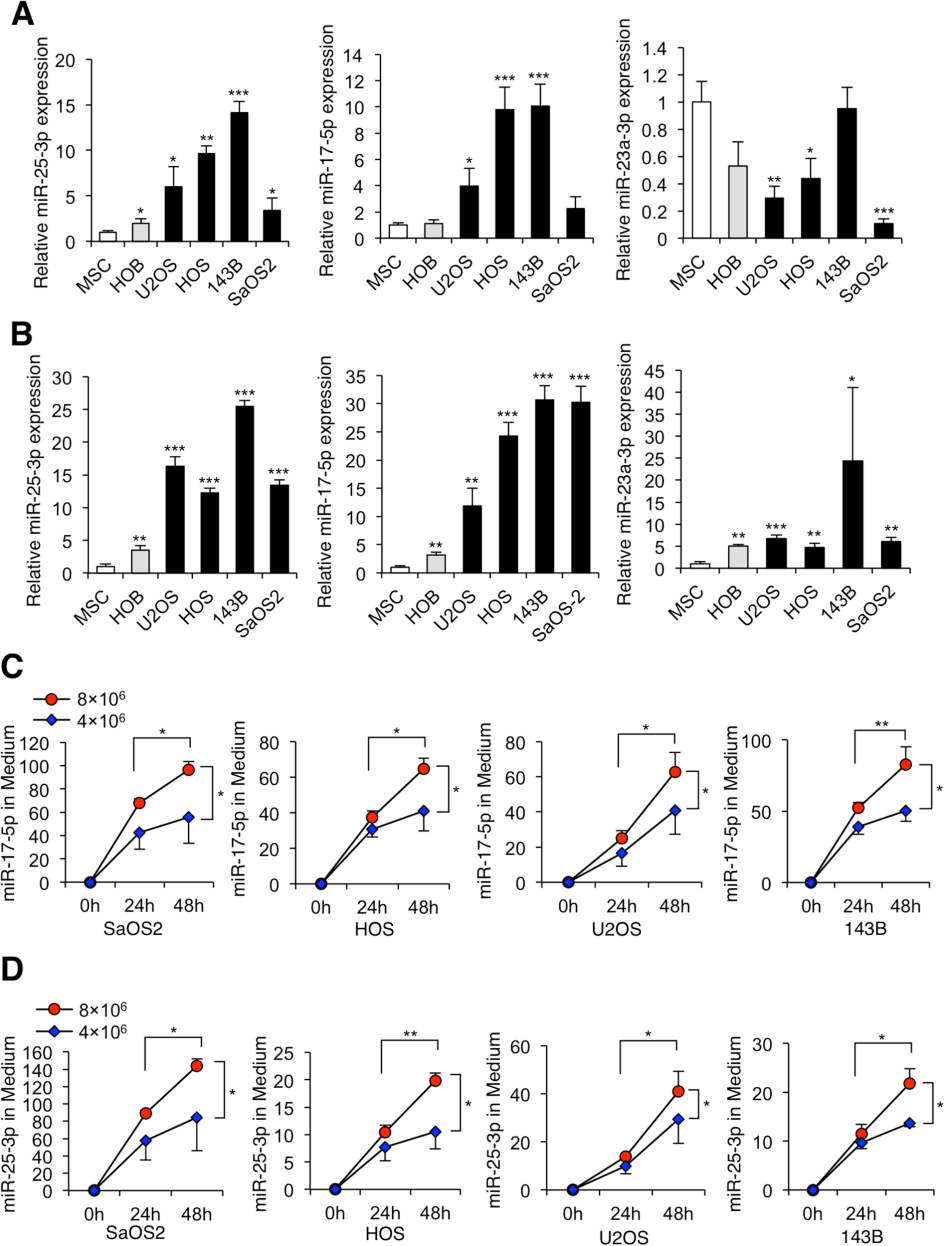
Secretory miRNAs from osteosarcoma cells **(A)** The expression levels of miR-25-3p, miR-17-5p, and miR-23a3-p in human osteosarcoma cells (SaOS2, HOS, U2OS, and 143B), human mesenchymal stem cells, and human osteoblast cells. Data are presented as means ± standard deviations (SD; n = 3 per group). *, *p* < 0.05; **, *p* < 0.01; ***, *p* < 0.001; Student's *t* test. **(B)** The expression of candidate miRNAs in the culture media of human osteosarcoma cells, human mesenchymal stem cells, and human osteoblast cells. Data are presented as means ± SD (n = 3 per group). *, *p* < 0.05; **, *p* < 0.01; ***, *p* < 0.001; Student's *t* test. **(C)** The expression of miR-17-5p in the culture media of osteosarcoma cell lines (SaOS2, HOS, U2OS, and 143B). The expression levels of miR-17-5p in the media of all cell lines increased with cell counts and longer incubation time. Data are presented as mean ± SD (n = 3 per group). *, *p* < 0.05; **, *p* < 0.01; ***, *p* < 0.001; one-way ANOVA with Bonferroni's multiple comparison. **(D)** The expression of miR-25-3p in the culture media of osteosarcoma cell lines. The expression levels of miR-25-3p in the media of all cell lines increased with cell counts and longer incubation time. Data are presented as mean ± SD (n = 3 per group). *, *p* < 0.05; **, *p* < 0.01; ***, *p* < 0.001; one-way ANOVA with Bonferroni's multiple comparison.

To determine whether these miRNAs acted in a secretory manner, we analyzed their expression levels in cultured human osteosarcoma cells and control normal cells, including human mesenchymal stem cells (MSCs) [52, 53] and HOB human osteoblast cells, as well as in culture media of these cells. qRT-PCR analyzes of RNA isolated from cell lines confirmed significant upregulation of miR-25-3p and miR-17-5p, but not other miRNAs (including miR-23a-3p), in both osteosarcoma cells and their culture media relative to normal control cells and their culture media (Figure 2A, 2B). Interestingly, the tendency of the expression levels of miR-25-3p and miR-17-5p between the cells and culture media were slightly different. Next, we determined whether miR-25-3p and miR-17-5p levels in the culture media were time course- and cell number-dependent. We observed that the expression levels of both miR-25-3p and miR-17-5p in the culture media from all seven cell lines increased with culture duration (24 and 48 h; *P* < 0.05) and with increasing numbers of tumor cells (*P* < 0.05) (Figure 2C, miR-17-5p; Figure 2D, miR-25-3p). These data suggest that miR-25-3p and miR-17-5p are secreted from human osteosarcoma cells.

### miR-25-3p and miR-17-5p expression levels in exosomes derived from osteosarcoma cells

In order to analyze whether circulating miR-25-3p and miR-17-5p are embedded in tumor-derived exosomes, we purified exosomes from the culture medium of osteosarcoma cell lines (U2OS, HOS, 143B, and SaOS2) and MSCs. We then analyzed the expression levels of exosomal and cellular miR-25-3p by qRT-PCR and compared expression ratios. Our results demonstrated that miR-25-3p was expressed to a greater extent in the exosomes compared with donor cells; this tendency was not observed in MSCs (Supplementary Figure 2).

### Expression profiles of serum miRNA levels during osteosarcoma development in tumor-bearing mice

**Figure 3:**
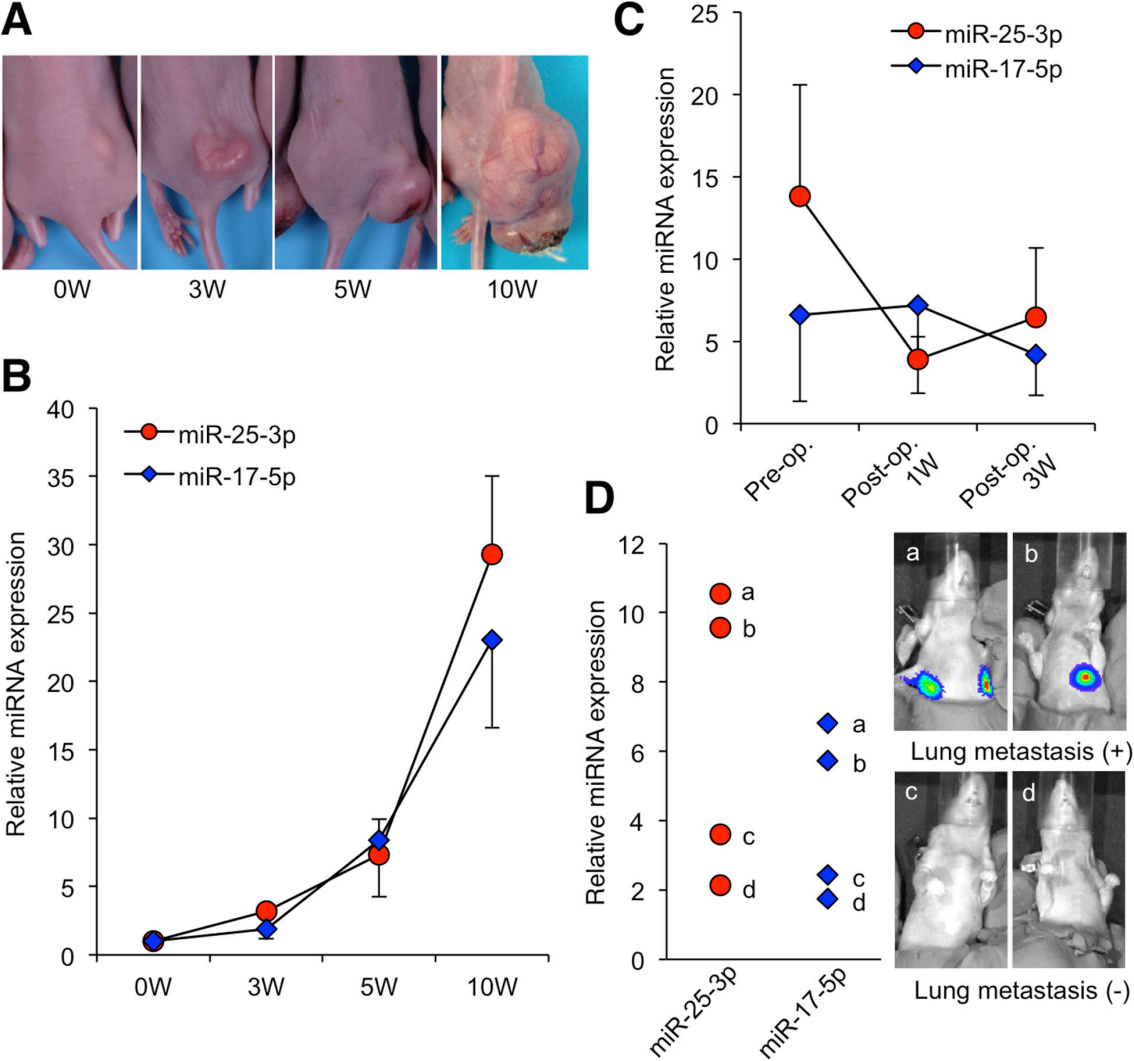
Serum miRNA levels during the development of osteosarcoma xenograft **(A)** The macroscopic appearances of 143B-luc tumors in each group of mice 0, 3, 5, and 10 weeks after tumor inoculation. **(B)** The medians of the relative expression levels of miR-17-5p and miR-25-3p plotted at 0, 3, 5, and 10 weeks. Data are presented as mean ± SD (n = 4 per group). **(C)** The medians of the relative expression levels of miR-17-5p and miR-25-3p plotted before and after (1 and 3 weeks) tumor resection (n = 4 per group). **(D)** The serum miR-17-5p and miR-25-3p levels of each mouse 3 weeks after tumor resection. The expression levels of miR-25-3p and miR-17-5p were higher in the serum of the two mice with lung metastasis.

To evaluate whether the serum expression levels of miR-25-3p and miR-17-5p could be used to monitor tumor dynamics *in vivo*, we examined the expression levels of these miRNAs in osteosarcoma-bearing mice. Serum miRNA expression levels were examined at different time points during tumor progression. Median serum levels in mice were plotted at day 0 before tumor cell inoculation and 3 weeks, 5 weeks, and 10 weeks after tumor cell inoculation. qRT-PCR analysis revealed that the expression levels of both miR-25-3p and miR-17-5p consistently increased with tumor development (Figure 3A, 3B). We also compared the expression levels of these miRNAs in pre- and post-operative states and identified that tumor resection decreased serum expression levels of miR-25-3p (Figure 3C). Among four mice with tumor resections, two developed lung metastasis 2 weeks after tumor resection. Interestingly, the expression levels of miR- 25-3p and miR-17-5p increased in the serum of the mice with lung metastasis (Figure 3D). These results suggest that these miRNAs reflect tumor burden in osteosarcoma-bearing mice.

### Evaluation of miR-25-3p and miR-17-5p expression levels in the serum of osteosarcoma patients

**Figure 4:**
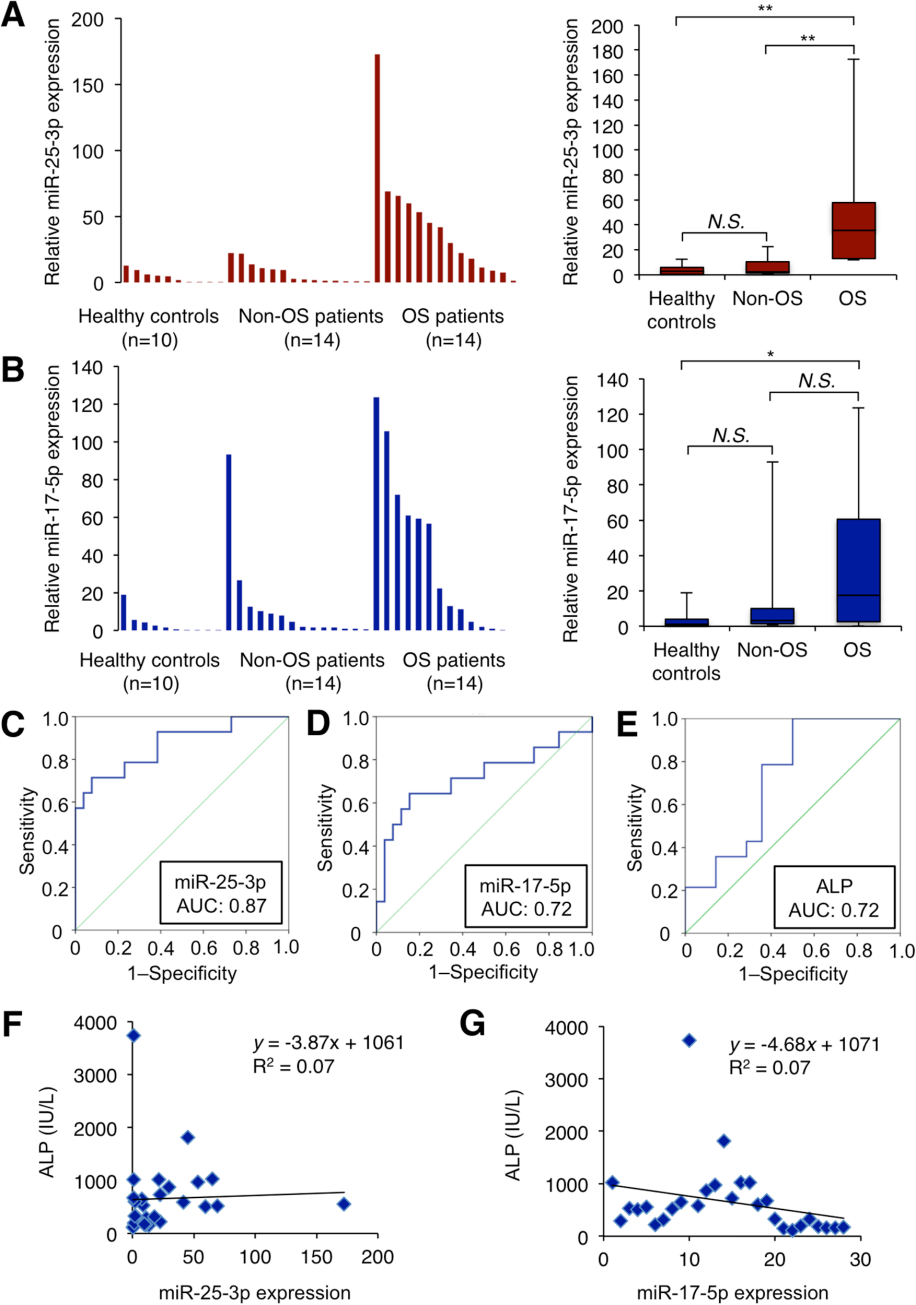
Serum miR-25-3p and miR-17-5p expression levels in the validation cohort of osteosarcoma patients **(A, B)** Serum miR-25-3p concentrations in 14 consecutive osteosarcoma patients, 14 age-matched non-osteosarcoma patients, and 10 healthy individuals. Serum miR-25-3p **(A)** and miR-17-5p **(B)** expression was detectable in all samples (left). Differential expressions of serum miR-25-3p and miR-17-5p in osteosarcoma patients were compared with those of controls by a waterfall plot (right). Concentrations of miR-25-3p were significantly higher in serum from osteosarcoma patients than in controls (*P* = 0.004). Concentrations of miR-17-5p were significantly higher in serum from osteosarcoma patients than in healthy volunteers (*P* = 0.021) but there was no statistical significance (*P* = 0.070) compared to the age-matched non-osteosarcoma patients. *, *P* < 0.05; **, *P* < 0.01; one-way ANOVA with Bonferroni's multiple comparison. **(C, D)** Receiver-operating characteristic (ROC) curve analysis of miR-25-3p and miR-17-5p for detecting osteosarcoma patients. ROC curve analysis showed the AUC of 0.868 (95% confidence interval, 0.743–0.993) for miR-25-3p **(C)** and 0.720 (95% confidence interval, 0.531–0.909) for miR-17-5p **(D)**, respectively. The analysis revealed that the sensitivity and specificity of miR-25-3p was 71.4% and 92.3%, respectively, whereas the sensitivity and specificity of miR-17-5p was 64.3% and 84.6%, respectively. **(E)** The results of ROC curve analysis of ALP. ROC analysis revealed that the sensitivity and the specificity was 100% and 50%, respectively, with an AUC of 0.724 (95% confidence interval, 0.530–0.919). **(F, G)** Correlation between serum miR-25-3p and miR-17-5p concentrations and serum ALP levels in osteosarcoma patients. There was no significant correlation between serum miRNA concentrations and ALP levels (Pearson correlation scatter plot).

We next evaluated serum expression levels of miR-25-3p and miR-17-5p by qRT–PCR in a validation cohort of 14 osteosarcoma patients, 14 age-matched non-osteosarcoma patients, and eight healthy controls. Serum concentrations of these two miRNAs in osteosarcoma patients were statistically compared with those in non-osteosarcoma patients and healthy volunteers. Serum concentrations of miR-25-3p in osteosarcoma patients were significantly higher than in non-osteosarcoma patients (*P* = 0.004) and healthy volunteers (*P* = 0.004) (Figure 4A). Serum concentrations of miR-17-5p in osteosarcoma patients were also significantly higher than healthy volunteers (*P* = 0.021). However, we did not identify a statistical difference in miR-17-3p levels between osteosarcoma patients and non-osteosarcoma patients (*P* = 0.070) (Figure 4B). Then, we further analyzed serum miR-25-3p levels in patients with chondrosarcoma and Ewing sarcoma, which are second and third most common types of primary bone sarcoma after osteosarcoma, and observed elevated serum miR-25-3p levels in patients with osteosarcoma relative to those with other sarcomas (Supplementary Figure 3).

Next, we performed receiver-operating characteristic (ROC) curve analysis to assess the potential usefulness of serum miR-25-3p and miR-17-5p as noninvasive biomarkers for osteosarcoma. ROC analyses revealed that serum miR-25-3p levels were robust in discriminating patients with osteosarcoma from non-osteosarcoma patients and healthy volunteers, with an AUC value of 0.868 (95% confidence interval = 0.743 to 0.993) (Figure 4C). Using a cutoff value determined by the Youden Index [54], the sensitivity and specificity values for identifying a patient with osteosarcoma were 71.4% and 92.3%, respectively. For miR-17-5p, the AUC value from ROC analysis based on the expression levels was 0.720 (95% confidence interval = 0.743 to 0.993), and sensitivity and specificity values were 64.3% and 84.6%, respectively (Figure 4D).

We also compared the AUC based on the expression levels of these miRNAs with that of ALP, a conventional biomarker for osteosarcoma [55]. Interestingly, the AUC value of miR-25-3p levels was higher than that of ALP (0.724, 95% confidence interval = 0.530 to 0.919) (Figure 4E). These results indicate that serum levels of miR-25-3p may be a useful biomarker for differentiating patients with osteosarcoma from those with non-osteosarcoma and healthy individuals. Indeed, there was no significant correlation between serum miR-25-3p or miR-17-5p concentrations and serum ALP (Figure 4F, 4G).

### Correlation between miR-25-3p and hematocytes in the patient blood

**Figure 5:**
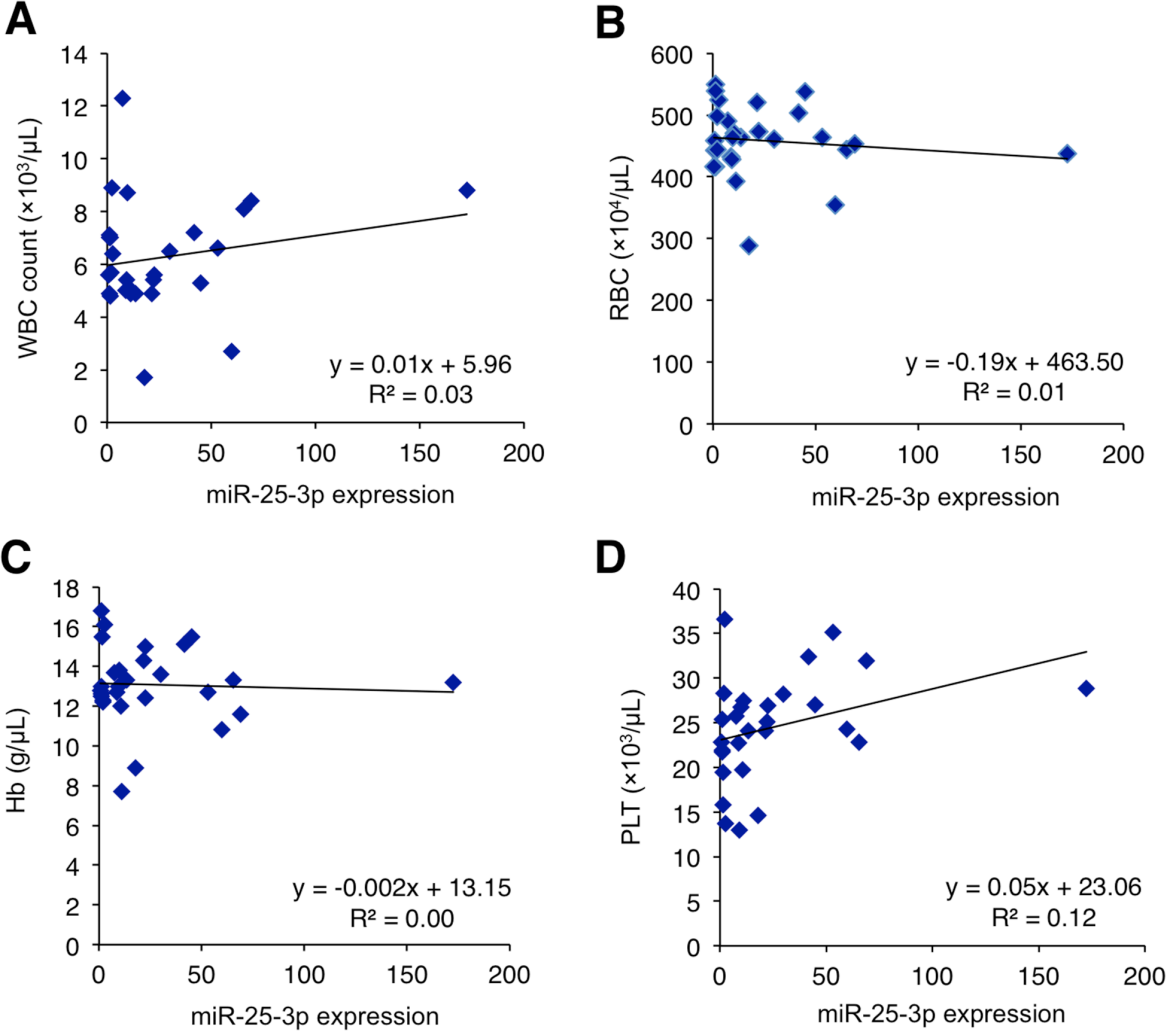
Correlation between serum miR-25-3p levels and the hematocytes of peripheral blood in the validation cohort of osteosarcoma patients **(A-D)** There was no significant correlation between serum miR-25-3p levels and any type of peripheral hematocytes (Pearson correlation scatter plot).

A recent report has cautioned that circulating miRNAs may reflect a blood-based phenomenon rather than a cancer-specific origin [56]. Accordingly, we analyzed the correlation between serum miR-25-3p and the hematocytes of peripheral blood in the validation cohort. No significant correlation between serum miR-25-3p concentrations and any type of hematocyte were observed (Figure 5A-5D). These results indicate that serum miR-25-3p may not be derived from hematocytes. Furthermore, serum miR-25-3p levels did not correlate with other data, including albumin (ALB), aspartate transaminase (AST), alanine transaminase (ALT), creatinine (Cr), and C-reactive protein (CRP) (Supplementary Figure 4).

### Clinicopathological evaluation of serum miR-25-3p expression levels in osteosarcoma patients

**Figure 6:**
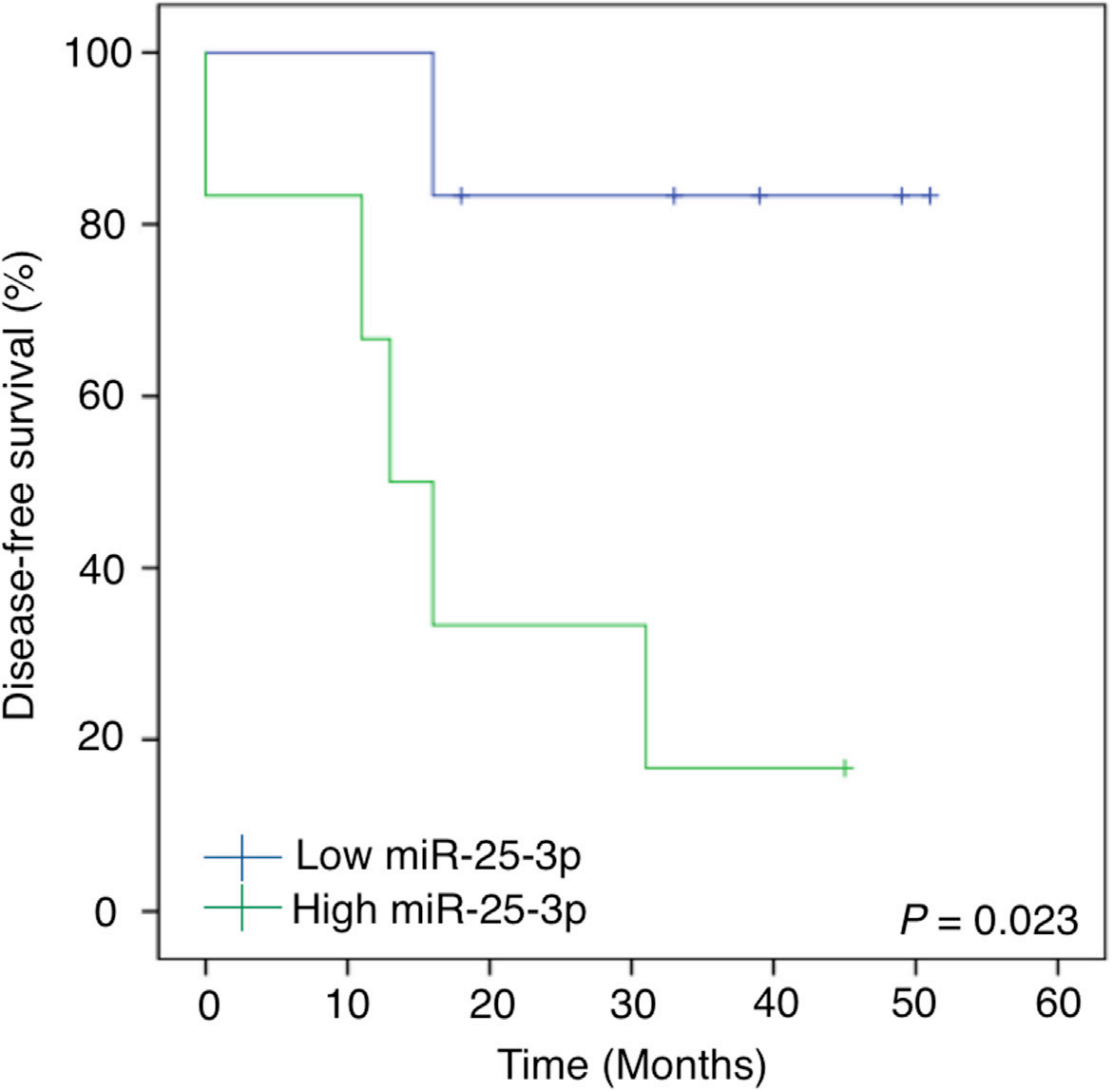
The prognostic value of serum miR-25-3p expression levels at diagnosis Kaplan-Meier analysis and log-rank test of disease-free survival according to serum miR-25-3p levels. The 3-year metastasis-free survival rate for patients with lower serum miR-25-3p levels was 83.3%, whereas that for patients with higher miR-25-3p levels was 16.7% (*P* = 0.023; log-rank test).

In order to evaluate the clinical relevance of miR-25-3p in the serum of osteosarcoma patients, we analyzed its prognostic significance with regard to various clinicopathological factors of patients who could be followed up in the validation cohort (Figure 6 and Table 1). An analysis of disease-free survival was performed using the Kaplan–Meier approach and the log-rank test. Patients were divided into two groups according to their serum miR-25-3p expression level by ROC curve analysis (Supplementary Figure 5). The prognostic value of miR-25-3p expression levels at diagnosis revealed a significant difference in the survival between patients with higher and lower expression levels of serum miR-25-3p (*P* = 0.023; Figure 6). The 3-year metastasis-free survival rate in patients with lower serum miR-25-3p expression was 83.3%, whereas that of patients with higher miR-25-3p expression was 16.7%. According to univariate analysis, we found that metastasis at diagnosis, distant metastasis, and serum level of miR-25-3p all significantly impacted survival (*P* = 0.001, *P* = 0.004, and *P* = 0.023, respectively). In contrast, age, gender, tumor site, and response to chemotherapy did not significantly impact survival (*P* = 0.454, *P* = 0.775, *P* = 0.403, and *P* = 0.932, respectively).

**Table 1:** Clinicopathological correlation with metastasis-free survival based on the univariate analysis in the validation cohort of osteosarcoma patients

Variable	Number of patients	Metastasis free 3-year survival (%)	*P* value(Log-rank test)
Age (years)			0.454
0–20	9	41.7	
21+	3	66.7	
Gender			0.775
Male	7	57.1	
Female	5	40.0	
Site			0.403
Femur	8	33.3	
Tibia	2	50.0	
Humerus	2	100.0	
Metastasis at diagnosis			0.001
Present	1	0.0	
Absent	11	53.0	
Distant metastasis during follow-up			0.004
Present	7	0.0	
Absent	5	100.0	
Response to neoadjuvant chemotherapy			0.932
Good (necrosis > 90%)	6	50.0	
Poor (necrosis < 90%)	6	50.0	
miR-25-3p expression			0.023
High	6	16.7	
Low	6	83.3	

### Evaluation of serum miR-25-3p expression for monitoring tumor dynamics in osteosarcoma patients

**Figure 7:**
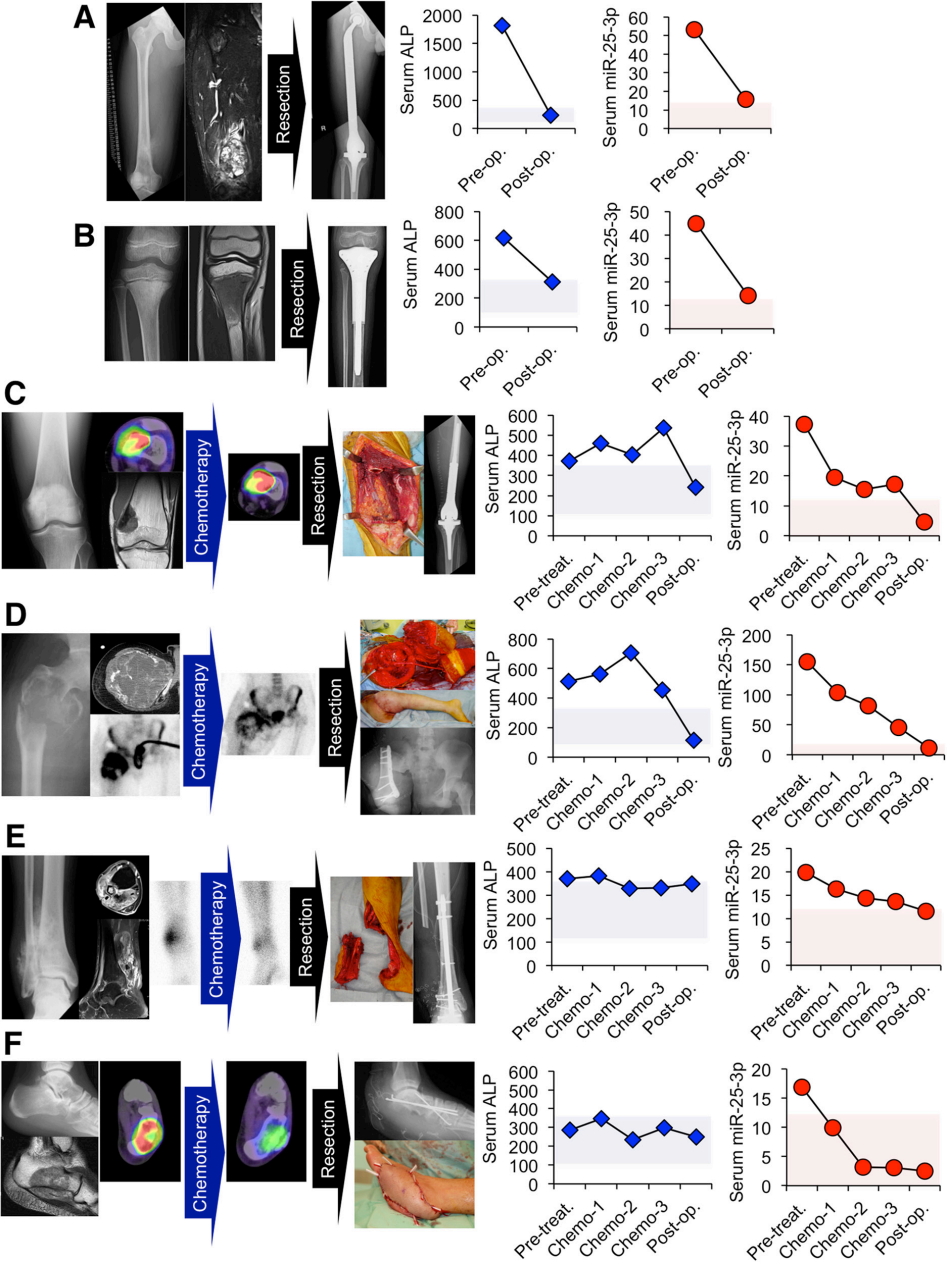
Tumor monitoring by serum miR-25-3p levels according to multi-modal treatment (**A, B)** Two cases whose serum ALP and miR-25-3p could be evaluated before and after surgical resection. Both serum ALP and serum miR-25-3p in a 17 year-old-male **(A)** and an 8 year-old-female **(B)** decreased after surgical resection. **(C-F)** Four cases whose serum ALP and miR-25-3p could be evaluated during neoadjuvant chemotherapy. **(C)** A 24 year-old-male with osteosarcoma in the distal femur. Tumor necrosis by neoadjuvant chemotherapy was 40%. Serum ALP levels increased during chemotherapy, while serum miR-25-3p levels decreased; both levels were reduced after tumor resection. **(D)** A 20 year-old-male with osteosarcoma in the proximal femur. Surviving tumor cells were not identified in the pathological evaluation after neoadjuvant chemotherapy. ALP levels did not change, but serum miR-25-3p levels decreased during chemotherapy. **(E)** A 50 year-old-male with osteosarcoma arising in the distal fibula. While serum ALP levels before surgical resection plateaued, serum miR-25-3p slightly decreased during chemotherapy. The pathological evaluation of the resected specimens reported 80% necrosis. **(F)** A 24 year-old-male with osteosarcoma in the calcaneous. Neoadjuvant chemotherapy reduced FDG accumulation and serum miR-25-3p levels also decreased during chemotherapy. ALP was within the normal range at all time points. Serum miR-25-3p levels corresponded with tumor necrosis, which was revealed as more than 95%. Pale blue; normal range of ALP, pale red; miR-25-3p levels below cut-off point.

To investigate whether serum miR-25-3p could monitor tumor dynamics in osteosarcoma patients, we evaluated serum miR-25-3p in several osteosarcoma patients according to the multi-modal treatment. Two cases (Case 1 and 2) could be monitored with their serum obtained at preoperative and postoperative status. Case 1 was a 17 year-old-male with osteosarcoma arising in the distal femur (Figure 7A). Both serum ALP and miR-25-3p expression decreased after tumor resection. Case 2 was an 8 year-old-female with osteosarcoma arising in the proximal tibia (Figure 7B). Similar to case 1, both serum ALP and miR-25-3p expression decreased after tumor resection. Four cases were monitored with their serum obtained at several points during treatment. Case 3 was a 24-year-old-male patient with osteosarcoma arising in the distal femur (Figure 7C). Neoadjuvant chemotherapy (high-dose methotrexate, doxorubicin, and cisplatin) did not reduce the SUVmax (11.22 to 20.81) and the subsequent 40% tumor necrosis rate in the resected specimens indicated a poor response. Serum ALP levels increased during chemotherapy (44%), whereas serum miR-25-3p levels decreased (54%), while both levels decreased after tumor resection. Regarding tumor necrosis, serum miR-25-3p levels, rather than serum ALP levels, appeared to correspond with the tumor burden. Case 4 was a 20-year-old male patient with a pathological fracture caused by osteosarcoma arising in the proximal femur (Figure 7D). Abnormal accumulation observed during thallium scintigraphy was reduced by neoadjuvant chemotherapy (high-dose methotrexate, doxorubicin, cisplatin, and ifosfamide), suggesting a favorable tumor response, and surviving tumor cells were not pathologically identified. Although his serum miR-25-3p levels decreased in response to neoadjuvant chemotherapy (70%), ALP levels did not change. Subsequently, serum miR-25-3p, rather than ALP, levels appeared to correlate with tumor necrosis. Case 5 was a 50-year-old male patient with osteosarcoma arising in the distal fibula (Figure 7E). Although decreased accumulation during thallium scintigraphy indicated a good tumor response to neoadjuvant chemotherapy (doxorubicin, cisplatin, and ifosfamide), the preoperative serum ALP levels plateaued. However, a slight decrease in serum miR-25-3p during chemotherapy (32%) was observed. Pathological evaluation of the resected specimens revealed 80% necrosis following chemotherapy, which correlated with a decrease in serum miR-25-3p, rather than ALP. Case 6 was a 24-year-old male patient with osteosarcoma arising in the calcaneous (Figure 7F). Neoadjuvant chemotherapy (high-dose methotrexate, doxorubicin, and cisplatin) reduced FDG-PET accumulation, indicating a good therapeutic response. Serum miR-25-3p levels also decreased during neoadjuvant chemotherapy (82%). However, ALP remained within the normal range at all time points and did not reflect drug response. Wide tumor resection did not reduce serum miR-25-3p levels; this finding corresponded with the >95% rate of tumor necrosis, suggesting that miR-25-3p might reflect viable cells and, therefore, drug response. Overall, data from these individualized cases suggest that serum miR-25-3p expression may be useful for tumor monitoring during multi-modal treatment.

## DISCUSSION

Clinical outcomes of osteosarcoma treatment have not significantly improved in over 20 years. To date, the most powerful predictors of outcome have remained the ability to detect metastatic disease at diagnosis and the histopathologic response of the tumor to neoadjuvant chemotherapy. Despite steady progress in the identification of genetic alterations in osteosarcoma, no individual molecular marker has been demonstrated to monitor drug response or have a better prognostic significance than the current clinical markers. Recent studies have identified that circulating mature miRNA in contrast to mRNA or snRNA is strikingly stable in the blood stream and cell culture medium [46, 47]. These circulating miRNAs were discovered in the peripheral blood of patients with malignant diseases and have provided novel insights into the biology of tumors and the effects of therapeutic interventions [46, 47].

To date, emerging evidence has begun to accumulate on miRNA dysregulation in osteosarcoma cells and/or tissues [26, 38]. Several studies have indicated that some of the miRNAs dysregulated in tumor tissue may exist in the blood samples of osteosarcoma patient [57–59]. Importantly, these studies have simply focused on the miRNA whose expression is dysregulated in osteosarcoma cells or tissues. However, there is no consensus whether or not miRNA dysregulation in tumor cells or tissues reflects those in the patient's blood. Indeed, recent investigations have demonstrated that miRNA profiling between tumor tissue and corresponding serum is largely dissimilar [60–62]. Pigati *et al*. performed miRNA microarray analysis of cellular and extracellular RNAs of MCF7, a breast cancer cell line, and found that approximately 66% of released miRNAs closely reflected cellular miRNAs [61]. Similarly, Chan et al. demonstrated that, among 20 miRNAs differentially expressed in breast cancer tumors compared with adjacent normal tissues, only seven miRNAs were upregulated in both tumors and serum [60]. These data suggest the existence of a cellular selection mechanism for miRNA release and indicates that released miRNAs do not necessarily reflect the abundance of miRNA in the cell of origin. Therefore, a circulating miRNA signature that shows favorable sensitivity and specificity could be detected by global expression analysis of patient's serum rather than the simple focus on dysregulated miRNAs in tumor cells or tissues. Indeed, this study, based on global miRNA screening, revealed that several oncogenic miRNAs in osteosarcoma cells and/or tissues such as miR-21 [63] and miR-214 [34, 64] were not detected as highly upregulated miRNAs in the patient serum, which is similar to other cancers [60–62]. Thus, this study is important as the first global miRNA screening of patient serum that detected circulating miRNA, with favorable sensitivity and specificity compared to the conventional marker.

In this study, global miRNA screening of patient serum, followed by *in vitro* analysis, detected miR-17-5p and miR-25-3p as secretory and circulating miRNAs in osteosarcoma patients. These miRNAs have also been reported as dysregulated miRNAs in osteosarcoma cells and/or tissues. The miR-17-92 cluster was among the first miRNAs to be validated as showing oncogenic potential [65, 66]. To date, the oncogenic function of miR-17-92 in osteosarcoma has been demonstrated by several researchers [24, 67–69]. On the other hand, upregulated miR-25 levels in osteosarcoma tissues compared with adjacent healthy tissues has been reported and its overexpression promoted cell proliferation and tumor growth [70]. In this study, we found that the sensitivity and specificity of serum miR-25-3p concentrations was superior to those of miR-17-5p. Notably, the relationship between the dysregulation of the miR-17-92 cluster and aging was reported [71], although the mechanistic connection between downregulation of the cluster's members and aging has yet to be elucidated [71]. Hackl *et al*. investigated different cell and tissue types representing aging and identified the downregulation of miR-17, miR-19b, miR-20a, and miR-106a [72]. Considering these reports, serum miR-25-3p expression levels would be a suitable non-invasive biomarker compared with serum miR-17-5p concentrations in osteosarcoma patients.

Various reports have shown that miR-25 plays an oncogenic role in esophageal cancer [73], cholangiocarcinoma [74], gastric cancer [75], and lung cancer [76], whereas other reports have indicated that miR-25 may function as a tumor suppressor in colon cancer and anaplastic thyroid carcinoma [77, 78]. Thus, whether miR-25 acts as an oncogene or a tumor suppressor may depend on the cellular context. Recent reports have shown that circulating miR-25 has diagnostic and/or prognostic value in patients with lung and hepatocellular cancer [79–81]. Therefore, this is the first evidence of the value of circulating miR-25 expression in mesenchymal malignancies, which enlarges the utility of this miRNA for various malignancies. In this study, we identified that there was no significant correlation between serum miR-25-3p concentrations and any type of hematocyte, which is consistent with other reports [56], while the use of whole blood may lead to the isolation of miRNAs from various cell types including those within the blood cells. Although Pitchard *et al*. suggested caution in the interpretation of circulating miRNAs as they may reflect a blood cell-based phenomenon rather than a cancer-specific origin, they demonstrated no correlation between miR-25, blood cells, and hemolysis [56]. Furthermore, while recent studies have demonstrated age-related miRNAs such as miR-21, miR-126-3p, miR-151a-5p, miR-181a-5p, and miR-1248 [71, 72, 82, 83], there has been no correlation of miR-25 and aging. Indeed, recent reports have demonstrated that miR-25 is upregulated in osteo-differentiated MSCs compared with MSCs [84], suggesting the correlation between miR-25 expression and bone metabolism, which may be different from ALP-related miRNAs including miR-27, miR-133, and miR-206 [85]. In this study, serum miR-25-3p concentration was a novel prognostic factor for osteosarcoma patients, which correlated with clinical distant metastasis. However, there was no statistical significance between the serum expression of this marker at diagnosis and drug sensitivity, as well as the other clinicopathological elements, in the validation cohort (Supplementary Table 4), which might be due to the small number of patients. An additional external validation based on a larger cohort is needed as the next step, which may yield clinically important information whether the detected serum miRNA could be a non-invasive biomarker of osteosarcoma.

Recent reports have demonstrated that most of the circulating miRNAs are included in lipid or lipoprotein complexes, such as apoptotic bodies, microvesicles, or exosomes, and are, therefore, highly stable [86]. In this study, we demonstrated that both miR-25-3p and miR-17-5p were enriched in the exosomes derived from osteosarcoma cell lines, indicating that they might be stable in the patient serum. One of the limitations of this study is that it was impossible to analyze the exosomal expression levels of miR-25-3p and miR-17-5p in patient serum because of the limited amount of serum that had been preserved. Indeed, the extraction procedures of exosomes from patient serum in clinical practice require additional time and efforts, which might inhibit the standardization of this methodology. Therefore, the simple procedure, without extraction of exosomes, could be practical for clinical application. On the other hand, liquid biopsies based on other methods, such as measurements of exosomes or circulating tumor cells (CTCs), have been reported. Suetsugu et al. successfully imaged tumor-derived exosomes in tumor-bearing mouse models by observing GFP-tagged CD63, a general exosomal marker, and found that these elements were incorporated into tumor-associated cells and circulated in the blood of mice with metastatic breast cancer [87]. Yoshioka et al. identified CD147 as a exosomal marker in colorectal cancer and demonstrated a sensitive and rapid ExoScreen-based analytical technique. Therefore, our future research plans include the use of tumor-derived exosomes as a novel methods of liquid biopsy. In addition, various trials have suggested the utility of CTCs as a liquid biopsy target. Shigeyasu et al. developed a method to capture live CTCs using a green fluorescent protein (GFP)-expressing telomerase-specific adenovirus and successfully imaged epithelial and mesenchymal tumor cells in clinical blood samples from patients with colorectal cancer [88]. Indeed, the roles of CTCs in tumor development have been evident based on the molecular imaging technologies in various malignancies including prostate, lung, and breast cancers [89–95]. To date, osteosarcoma CTCs have been detected by several authors [96] but have not been applied for liquid biopsy, which represents a future challenge.

In this study, we compared serum miRNA levels before and after tumor resection in a subcutaneous xenograft osteosarcoma model. To date, various reports have described molecular mechanisms of sarcoma metastasis based on orthotopic sarcoma models [97–100]. Indeed, we initially used an orthotopic osteosarcoma model. However, the orthotopically xenografted mice died after tumor resection (amputation) following complications such as bleeding or infection. In contrast, all mice with subcutaneous xenografts remained alive after tumor resection. Therefore, subcutaneous tumor inoculation might be less harmful and appropriate if the study protocol includes tumor resection and subsequent observation. As no reports have described differences in miRNA expression between orthotopic and non-orthotopic models, secretory miRNA profiling of *in vivo* osteosarcoma models would be an interesting step toward elucidating the roles of secretory miRNA in tumor tissue–microenvironment interactions.

In conclusion, serum-based miRNA signatures associated with osteosarcoma were successfully documented and validated. miR-25-3p was secreted from the established osteosarcoma cell lines and detected in tumor-derived exosomes. Serum miR-25-3p levels were upregulated in osteosarcoma patients and showed favorable sensitivity and specificity, correlating with poor prognosis. Furthermore, these concentrations closely correlated with tumor burden in animal models as well as with the therapeutic status of osteosarcoma patients. The clinical development of this methodology as a noninvasive diagnostic or monitoring strategy may be promising for clinicians and patients with malignant diseases.

## MATERIALS AND METHODS

### Patients and samples

Blood samples were collected from osteosarcoma patients, age-matched non-osteosarcoma patients with other benign tumors including enchondroma, non-ossifying fibroma, solitary bone cyst, lipoma, and hemangioma, and healthy volunteers at the National Cancer Center Hospital and Okayama University Hospital. Patient clinical information is summarized in Supplementary Tables 2, 3. Post-operative samples from three patients were obtained at 1–2 weeks after tumor-wide resection. Post-chemotherapy samples were obtained at the end of the cource. Serum samples were stored at −80°C until further processing. All patients provided written, informed consent authorizing the collection and use of their samples for research purposes. The study protocol was approved by the Institutional Review Board of the National Cancer Center Hospital and Okayama University Hospital.

### Cell lines and cell culture

The human osteosarcoma cell lines SaOS2, U2OS, HOS, and 143B were purchased from the American Type Culture Collection (ATCC). Human mesenchymal stem cells and human osteoblast cell line (HOB) were purchased from Takara Bio. The human osteosarcoma cell line 143B-luc was previously established in our laboratory [101]. We cultured SaOS2 in RPMI 1640 (Life Technologies). U2OS, HOS, 143B, and 143B-luc cells were cultured in DMEM (Life Technologies). All media were supplemented with 10% heat-inactivated FBS (Life Technologies), penicillin (100 U/mL), and streptomycin (100 mg/mL). Cells were maintained under 5% CO2 in a humidified incubator at 37°C.

### Isolation of exosomes

Conditioned medium was collected from 48-h cell cultures and centrifuged at 2,000 x g for 10 min, followed by filtration through a 0.22-μm filter to remove cell debris. The conditioned medium was then used for exosome isolation. Exosomes were harvested by ultracentrifugation at 110,000 x g for 70 min at 4°C. Pellets were washed with phosphate-buffered saline (PBS), subject to ultracentrifugation, and resuspended in PBS.

### RNA extraction

Total RNA was extracted from 800 μL of serum and culture media samples using miRNeasy RNA isolation Kits (Qiagen). Total RNA was isolated from tissue specimens, cultured cells, and exosomal pellets using the TRIzol reagent (Invitrogen), followed by homogenization by pipetting up and down several times. To allow for normalization of sample-to-sample variation, 25 fmol of synthetic *C. elegans* miRNA cel-miR-39 (5 μL of 5 nM miRNA mimic) (Qiagen) was added to the denatured samples. Total RNA in serum samples was then extracted according to the manufacturer's protocol.

### miRNA array

Global miRNA expression profiling was performed using a miRNA microarray manufactured by Agilent Technologies (Santa Clara). Labeling and hybridization of total RNA samples were performed according to the manufacturer's protocol. Each microarray experiment used 2 ng of miRNA containing total RNA. The microarray results were extracted using Agilent Feature Extraction software (v10.7.3.1) and analyzed using GeneSpring 12.6.1 software (Agilent Technologies).

### Quantitative reverse-transcription polymerase chain reaction

The expression levels of miRNAs in osteosarcoma cells were analyzed by TaqMan quantitative real-time PCR (TaqMan MicroRNA Assays; Applied Biosystems) using a 7300 Real-Time PCR system (Applied Biosystems) according to the manufacturer's protocol. Relative expression was calculated using the 2–^ΔΔCt^ method [102].

### Animal experiments

The animal experiments in this study were performed in compliance with the guidelines of the Institute for Laboratory Animal Research at Okayama University. Athymic nude mice (CLEA Japan) were purchased at 4 weeks of age and given at least 1 week to adapt to their new environment prior to tumor transplantation. On day 0, the mice were anesthetized with 3% isoflurane, and the right leg was disinfected with 70% ethanol. A 100 μL volume of solution containing 143B-luc cells (1 × 10^6^) was subcutaneously injected. The size of tumor growth was monitored, and whole blood was taken by cardiac puncture from mice at different times (3, 5, and 10 weeks after tumor transplantation) or after tumor resection. Blood samples were allowed to stand at room temperature for at least 1 h and for a maximum of 2 h. Mouse serum was separated from clots by centrifugation at 3500 rpm for 15 min at 4°C and stored at −80°C.

### Statistical analysis

All statistical analyses were performed using the SPSS statistical software package (version 23; SPSS Inc.). Student's *t*-test or one-way ANOVA, corrected for multiple comparisons as appropriate, were used to compare differences in cellular, secretory, and serum miRNA concentrations and miRNA ratios between the osteosarcoma, age-matched control, and healthy control groups. Receiver-operating characteristic (ROC) curves and area under the curve (AUC) were used to assess the feasibility of using serum miRNA concentrations as a diagnostic tool for detecting osteosarcoma. The Younden Index was used to determine the cutoff value for serum miRNA concentrations [103]. The Kaplan–Meier method and the log-rank test were used to compare the survival of patients. For all analyses, we considered a *P* value of 0.05 or less to be significant.

## SUPPLEMENTARY MATERIALS FIGURES AND TABLES


