# Supplementary material for: Clinical significance of circulating miR-25-3p as a novel diagnostic and prognostic biomarker in osteosarcoma
Source: Oncotarget. 2017 Mar 23;8(20):33375–92. doi: 10.18632/oncotarget.16498 (PMC5464875; doi:10.18632/oncotarget.16498)
Supplement: Supplementary file 1 [file oncotarget-08-33375-s001.pdf]

Clinical significance of circulating miR-25-3p as a novel diagnostic and prognostic biomarker in osteosarcoma

SUPPLEMENTARY FIGURES AND TABLES

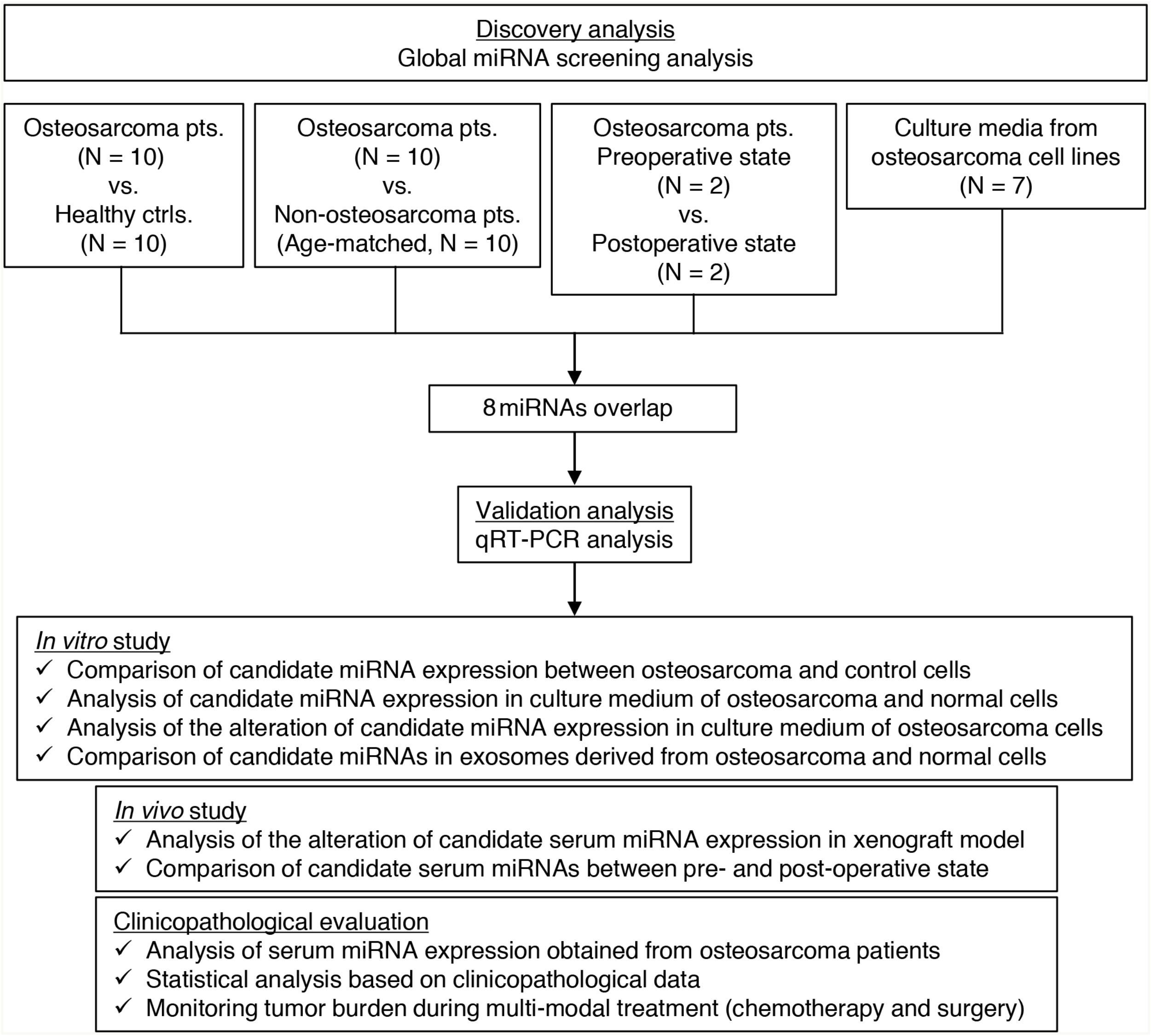

Supplementary Figure 1: Study design. Global miRNA screening analysis was performed using sera obtained from 10 osteosarcoma patients, 10 age-matched benign tumor patients, and 10 healthy controls, as well as culture media from 7 osteosarcoma cell lines. Additional sets were used for other clinical validations, followed by *in vitro*, *in vivo* study, and clinicopathological evaluation

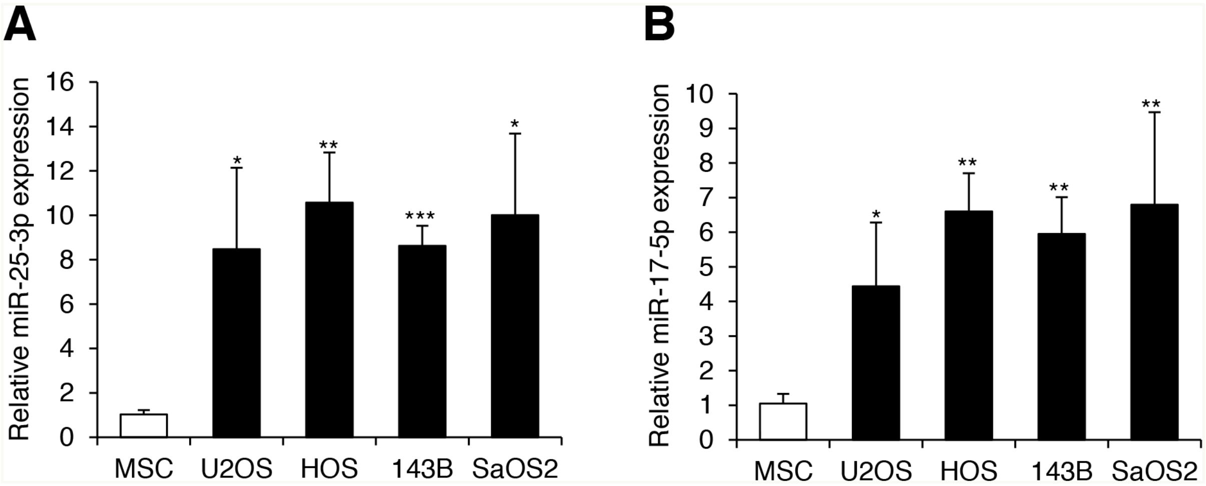

Supplementary Figure 2: Exosomal miR-25-3p and miR-17-5p levels in culture media of osteosarcoma cells. **(A, B)** The ratios of exosomal to cellular miR-25-3p **(A)** and miR-17-5p **(B)**. Exosomal miRNAs and cellular miRNAs expression were evaluated, and the ratios of exosomal to cellular miRNAs were caluculated. In all osteosarcoma cell lines we used, both miR-25-3p and miR-17-5p were found to be expressed to a greater extent in the exosomes compared with their donor cells, whereas it was not in the control mesenchymal stem cells. *, $p < 0.05$; **, $p < 0.01$; ***, $p < 0.001$; Student's *t* test.

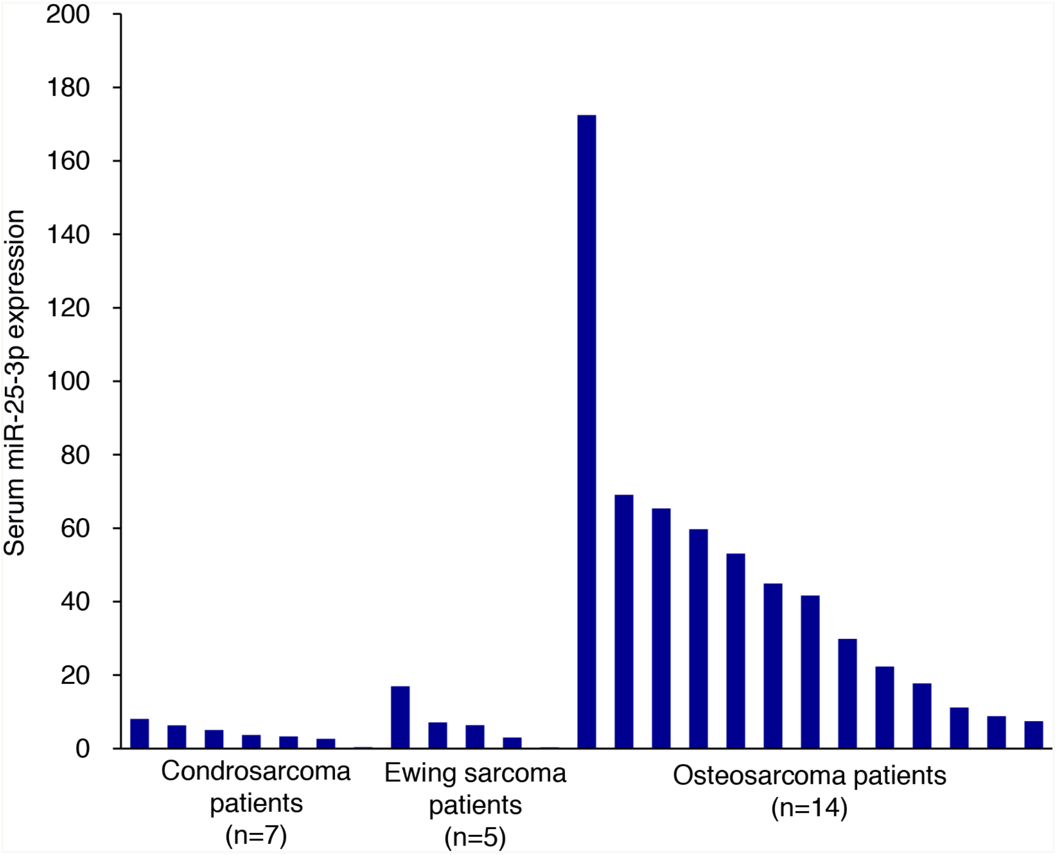

Supplementary Figure 3: Serum miR-25-3p expression levels in patients with osteosarcoma, Ewing sarcoma, and chondrosarcoma. Elevated serum miR-25-3p levels were observed in patients with osteosarcoma, the most common type of primary bone sarcoma, relative to those with chondrosarcoma and Ewing sarcoma, the second and third most common types of primary bone sarcoma.

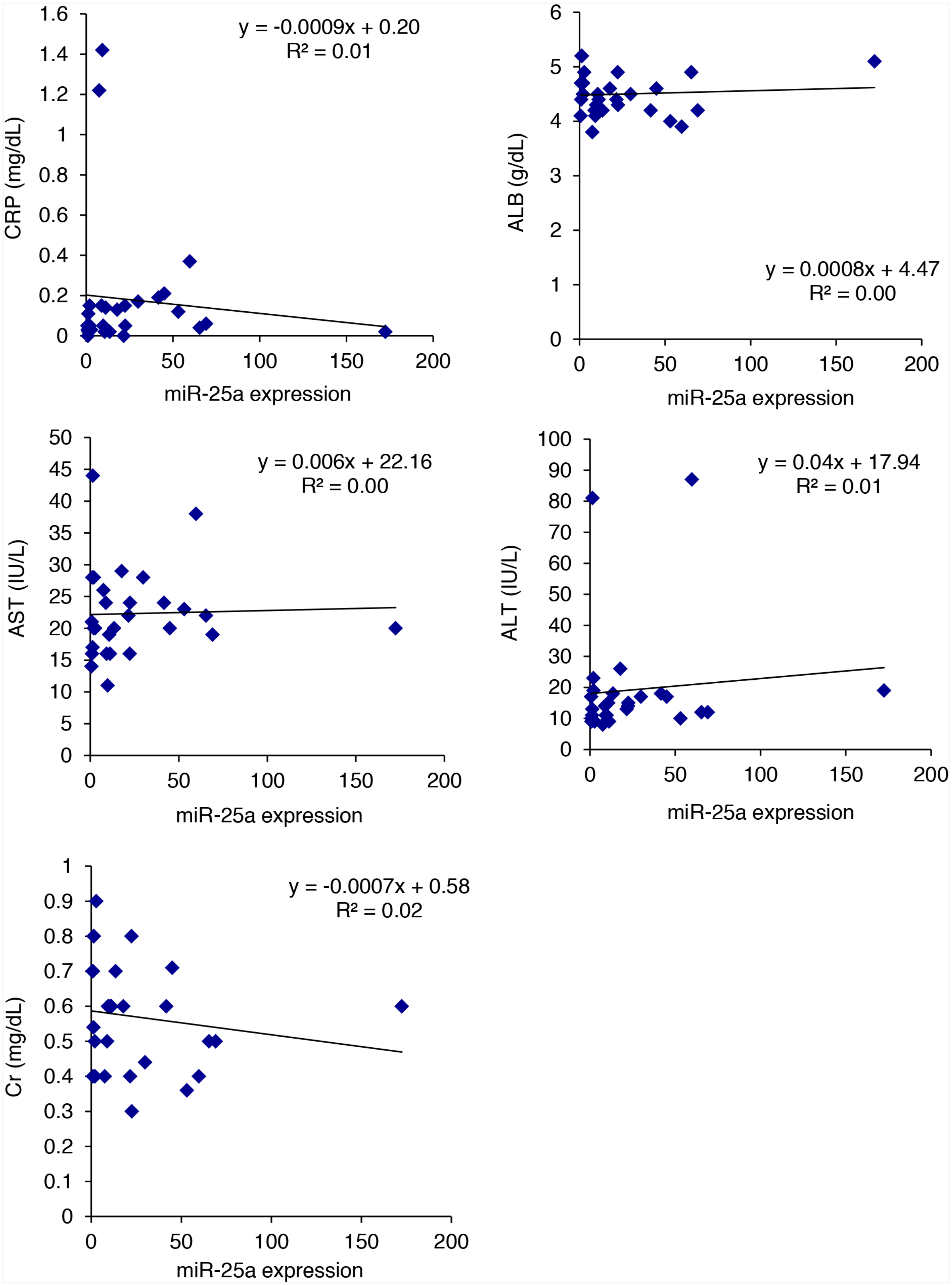

Supplementary Figure 4: Correlation between serum miR-25-3p levels and the blood chemistry assessments of peripheral blood in osteosarcoma patients. There was no significant correlation between serum miR-25-3p concentrations and the levels of albumin (ALB), aspartate aminotransferase (AST), alanine aminotransferase (ALT), creatinine (Cr), and C-reactive protein (CRP) (Pearson correlation scatter plot).

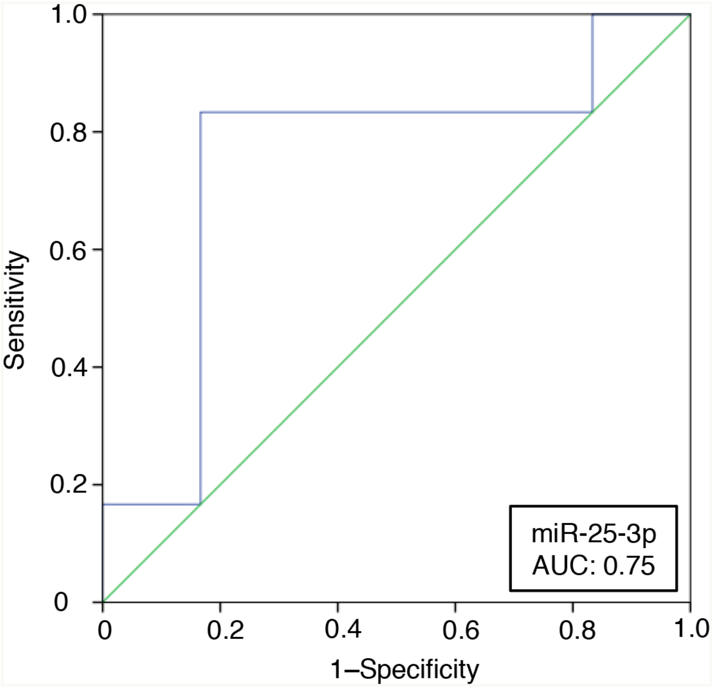

Supplementary Figure 5: ROC curve for serum expression of miR-25-3p in the validation cohort. The area under the ROC curve was 0.750. The cutoff was set at the point representing 83.3% sensitivity and 83.3% specificity.

Supplementary Table 1: Up-regulated miRNAs in the serum of osteosarcoma patients compared to control individuals

Systematic_name	Fold Change (OS vs CONT)	Fold Change (OS vs NON)	Regulation	*P* value
hsa-miR-24-3p	135.74	2.81	up	0.00008
hsa-miR-23a-3p	51.41	2.25	up	0.0007
hsa-miR-17-5p	44.29	2.25	up	0.0006
hsa-let-7i-5p	26.37	2.24	up	0.0001
hsa-miR-25-3p	14.73	2.13	up	0.0003
hsa-miR-92a-3p	7.61	1.73	up	0.000006
hsa-miR-1268a	2.41	1.71	up	0.002
hsa-miR-6087	1.72	1.78	up	>0.05

Supplementary Table 2: Clinicopathological characteristics of osteosarcoma patients investigated in the discovery and validation cohort

Variable	Discovery cohort	Validation cohort
Age (years)		
0–10	1	2
11–20	5	8
21+	4	4
Gender		
Male	4	7
Female	6	7
Site		
Femur	6	10
Tibia	2	2
Humerus	2	2
Metastasis at diagnosis		
Present	1	1
Absent	9	13
Neoadjuvant chemotherapy		
MTX+DOX/CDDP	6	8
MTX+DOX/CDDP+IFO	4	6
Response to neoadjuvant chemotherapy		
Good (necrosis > 90%)	3	6
Poor (necrosis < 90%)	5	6
NA	2	2
Disease status		
NED	6	7
AWD	3	4
DOD	1	3

MTX, methotrexate; DOX, doxorubicin; CDDP, cisplatin; AWD, alive with disease; NED, no evidence of disease ; DOD, dead of disease; IFO, ifosfamide; NA, not available.

Supplementary Table 3: Patients that are monitored for serum miR-25-3p during treatment

Case No.	Age	Sex	Site	Prox./ dist.	Subtype	UICC stage	Neoadju. chemo	Drug Response	Surgery	Rec.	Mets.	Follow-up (months)	Disease status
1	17	M	Femur	Dist.	Conventional	III	MAP	Poor	Wide resection	-	Lung	20	DOD
2	8	F	Tibia	Prox.	Conventional	IIA	MAP	Poor	Wide resection	-	Lung	32	AWD
3	17	M	Femur	Dist.	Conventional	III	AP+I	Poor	Wide resection	-	-	20	NED
4	20	M	Femur	Prox.	Telangiectatic	IIB	MAP+I	Good	Wide resection	-	-	11	NED
5	51	M	Fibula	Dist.	Dedifferentiated	IIB	MAP	Poor	Wide resection	-	-	16	NED
6	25	M	Calcaneous	-	Chondroblastic	IIA	MAP	Good	Wide resection	-	-	18	NED

M, methotrexate; A, adriamycin; P, cisplatin; I, ifosfamide; DOD, dead of disease; AWD, alive with disease; NED, no evidence of disease.

Supplementary Table 4: Clinicopathological correlation based on miR-25-3p expression in the validation cohort of osteosarcoma patietns

Variable	Number of patients	Low miR-25-3p	HighmiR-25-3p	Correlation
Age (years)				0.091
0–20	9	3	6	
21+	3	3	0	
Gender				0.5
Male	7	4	3	
Female	5	2	3	
Site				0.135
Femur	8	4	4	
Tibia	2	0	2	
Humerus	2	2	0	
Metastasis at diagnosis				0.5
Present	1	0	1	
Absent	11	6	5	
Distant metastasis during follow-up				0.121
Present	7	2	5	
Absent	5	4	1	
Response to neoadjuvant chemotherapy				0.284
Good (necrosis > 90%)	6	4	2	
Poor (necrosis < 90%)	6	2	4	
Disease status				0.341
NED	6	4	2	
AWD	3	1	2	
DOD	3	1	2	
miR-25-3p expression				–
High	6	0	6	
Low	6	6	0	

NED, no evidence of disease; AWD, alive with disease; DOD, dead of disease.
